# Hinokitiol impedes tumor drug resistance by suppressing protein kinase B/mammalian targets of rapamycin axis

**DOI:** 10.7150/jca.69449

**Published:** 2022-03-14

**Authors:** Ying-Jui Ni, Zi-Ni Huang, Hsin-Yu Li, Chiao-Ching Lee, Yu-Chang Tyan, Ming-Hui Yang, Christian R. Pangilinan, Li-Hsien Wu, Yu-Chung Chiang, Che-Hsin Lee

**Affiliations:** 1 Division of Gastroenterology, Department of Internal Medicine, Kaohsiung Armed Forces General Hospital, Kaohsiung, Taiwan.; 2 Department of Biological Sciences, National Sun Yat-sen University, Kaohsiung 80424, Taiwan.; 3 Department of Medical Imaging and Radiological Sciences, Kaohsiung Medical University, Kaohsiung, Taiwan.; 4 Department of Medical Education and Research, Kaohsiung Veterans General Hospital, Kaohsiung, Taiwan.; 5 Department of Medical Research, China Medical University Hospital, China Medical University, Taichung 404, Taiwan.; 6 Department of Medical Laboratory Science and Biotechnology, Kaohsiung Medical University, Kaohsiung 80708, Taiwan.; 7 International PhD Program for Science, National Sun Yat-sen University, Kaohsiung 80424, Taiwan.; 8 Aerosol Science Research Center, National Sun Yat-sen University, Kaohsiung, Taiwan, 80424, Taiwan.

**Keywords:** Hinokitiol, P-glycoprotein, 5-Fluorouracil, tumor, combination therapy

## Abstract

Chemotherapy is a treatment method commonly used for cancer and that patients showing low to no response to the treatment often developed drug resistance via multiple mechanisms. Natural products have been shown to reduce tumor drug resistance. Hinokitiol, a natural tropolone derivative, has potential as an antitumor agent. To improve the efficacy and safety of hinokitiol, a further understanding of hinokitiol interactions with the tumor microenvironment is necessary. The presence of plasma membrane multidrug resistance protein P-glycoprotein (P-gp) is favorable for tumor cells to elicit chemotherapeutic resistance. Here, we showed that hinokitiol dose-dependently decreased P-gp expression and suppressed the P-gp-driven efflux activity based on Rhodamine 123 assay. The protein expression levels of phosph-protein kinase B (P-AKT), phosph-mammalian targets of rapamycin (P-mTOR), and phosph-p70 ribosomal s6 kinase (P-p70s6K) in tumor cells were likewise reduced after hinokitiol treatment. The transfection of cells with active P-AKT rescued hinokitiol-induced downregulation of P-gp, suggesting the involvement of Akt/mTOR/p70s6K signaling in P-gp expression. Our results showed that hinokitiol can chemosensitize cancer cells. These findings indicate that hinokitiol could enhance 5-Fluorouracil therapeutic effects in murine B16F10 and CT26 tumor cells via downregulation of the AKT/mTOR pathway.

## Introduction

The development of drug resistance is a major hindrance in cancer treatment that ultimately leads to relatively low patient survival in many cancer types 1. Drug resistance takes place when cancer cells increase drug efflux, reduce drug intake, disrupt cell cycle, and/or alter the drug target, among other known mechanisms 2. One of the well-studied resistance mechanisms is the substantial increase in drug efflux activity driven by high expression of ATP-binding cassette (ABC) transporters. For instance, P-glycoprotein (P-gp)—belonging to the family of ABC transporters—prevents intracellular accumulation of chemotherapeutic drugs via efflux potential and therefore significantly reduces the efficacy of drugs 3,4. In fact, several reports demonstrated that P-gp-mediated drug efflux is one of the clinically relevant phenomena that drives resistance in many cancers 5,6. Reversing drug resistance by blocking or inhibiting P-gp has been shown to increase cellular retention of chemotherapeutics accompanied by an increase in tumor cell death 7,8.

A recent review on plant-derived compounds suggests the potential of natural products in overcoming drug resistance that offers safety and potential synergistic effects in chemotherapy 9. Hinokitiol (β-thujaplicin) is a bioactive compound, isolated from the heartwood of cupressaceous Taiwanese hinoki, with diverse pharmacological and biological activities 10. Hinokitiol can be used in the oral, skin care products, and food additives. Hinokitiol has been demonstrated to prompt a wide range of biological activities, including anti-fungal, anti-bacterial and anti-tumor activities 11. The effects of hinokitiol against tumor cells involves activation of apoptosis and autophagy resulting in tumor growth retardation 12, 13. Further, hinokitiol has been shown to inhibit cancer cell through cell-cycle arrest and the downregulation of X-linked inhibitor of apoptosis 14, 15. Hinokitiol possesses the ability to reduce tumor growth without inducing toxicity in normal cells. Dependent on the beneficial effects of hinokitiol, the synergistic action of hinokitiol with known cancer drugs is worth studying. Here, we used 5-Fluorouracil (5-FU)—a drug widely used for the treatment of malignant tumors—to determine whether hinokitiol can overcome drug efflux via downregulating P-gp thereby improving 5-FU tumoricidal efficacy. 5-FU has a relatively narrow therapeutic window and has been observed to have toxicity to normal cells 16. The important dose-limiting factor of 5-FU is leukopenia. Leukopenia can cause blood toxicity and increased risk to infectious diseases. The bone marrow cell apoptosis has been recognized as the mechanism for 5-FU-induced leukopenia 17. Considering the side effects induced by 5-FU and emergence of multidrug resistance, there is an urgent need in optimizing treatment for tumors. It is a good strategy to improve the sensitivity of tumor cells to chemotherapy or reduce the drug-resistance of tumor cells. The potential application of hinokitiol as chemosensitizer has not been well studied. Our results validate hinokitiol as a potent natural compound that exerts powerful effects in inducing chemosensitivity.

## Material and Methods

### Reagents, cell lines, plasmid, and animals

Hinokitiol (purity: 99%), rhodamine 123 (Rho-123), 5-Fluorouracil (5-FU) and dimethyl sulfoxide (DMSO) were purchased from Sigma-Aldrich (Sigma Aldrich, St. Louis, MO, USA). Dr. Chiau-Yuang Tsai (Department of Molecular Immunology, Osaka University, Japan) provided the constitutively active AKT plasmid 18. The B16F10 (mouse melanoma) 18 and CT26 (mouse colon cancer) 19 cells were cultured in Dulbecco's Modified Eagle Medium (DMEM) containing 1% antibiotics (100 units/mL penicillin and 100 μg/mL streptomycin), 2mM l-glutamine and 10% fetal bovine serum (FBS). The C57BL/6 (B16F10 mouse tumor model) and BALB/c (CT26 mouse tumor model) female mice were purchased from the National Laboratory Animal Center of Taiwan. The experimental protocol adhered to the rules of the Animal Protection Act of Taiwan and was approved by the Laboratory Animal Care and Use Committee of the National Sun Yat-sen University.

### Cell viability

Tumor cells were treated with varying concentrations of hinokitiol for 16 h (0, 1.25, 12.5, 125 and 1250 μM). Cell viability was assessed by colorimetric WST-8 assay (Dojindo Labs, Tokyo, Japan) according to the manufacturer's instructions.

### Rhodamine (Rho)-123 assay

Tumor cells (B16F10 or 4T1) were plated in culture plates and treated with various concentrations of hinokitiol for 16 h. The P-gp functional activity in B16F10 and CT26 cells was measured by the retention of Rho-123. The accumulation of Rho-123 in cells was also analyzed using spectrometer (BMG Labtech, Headquarters Germany) and a fluorescence microscope 8.

### Apoptotic assay

The tumors were excised and snap frozen at day 15. Terminal deoxynucleotidyl transferase (TdT) dUTP nick end labeling assay (TUNEL) assay was used to detect cell apoptosis within tumors and was performed according to the manufacturer's instructions (Promega, Madison, WI, USA). TUNEL-positive cells (brown staining) were counted under the microscope. We counted three high-power (× 200) fields with approximately 400-450 cells that showed highest density of positive-stained cells per field to determine the average percentage of apoptotic (TUNEL positive) cells in each section.

### Immunoblot analysis

The protein content was determined using bicinchoninic acid (BCA) protein assay (Pierce Biotechnology, Rockford, IL, USA). About 60-80 µg of protein from the lysates were loaded and fractionated on SDS-PAGE, transferred onto Hybond enhanced chemiluminescence nitrocellulose membranes (Amersham, Little Chalfont, UK) and detected with antibodies against P-gp (GeneTex, Inc. Irvine, CA, USA), the mammalian target of rapamycin (mTOR) (Cell Signaling, Danvers, MA, USA), phosph-mTOR (Cell Signaling), protein kinase B (AKT) (Santa Cruz Biotechnology, Inc. Santa Cruz, CA, USA), phosph-AKT (Santa Cruz Biotechnology, Inc.), p70s6K (Cell Signaling), phosph-p70s6K (Cell Signaling), caspase 3 (Cell Signaling), β-actin (Sigma Aldrich). Rabbit anti-mouse IgG-peroxidase antibody (Sigma Aldrich) and goat anti-rabbit IgG-peroxidase antibody (Sigma Aldrich) were used as the secondary antibody. The protein-antibody complexes were visualized using enhanced chemiluminescence system. The Western blotting signals were quantified with ImageJ software (rsbweb.nih.gov/ij/) 20.

### Animal study

Mice were inoculated subcutaneously (s.c.) with 10^6^ B16F10 or CT26 cells at day 0, and tumor-bearing mice were injected intraperitoneally (i.p.) with hinokitiol (40 mg/kg) at day 7 followed by 5-FU (40 mg/kg) treatment on days 9, 11, and 13. A separate set of mice for hinokitiol or 5-FU without combinatorial treatment were also prepared. All mice were monitored for tumor growth as previously described 21.

### Statistical analysis

The one-way analysis of variance (ANOVA) was used to determine differences among experimental groups. A P value of less than 0.05 is regarded as statistically significant.

## Results

### Cell viability in B16F10 and CT26 following hinokitiol treatment

**Figure 1:**
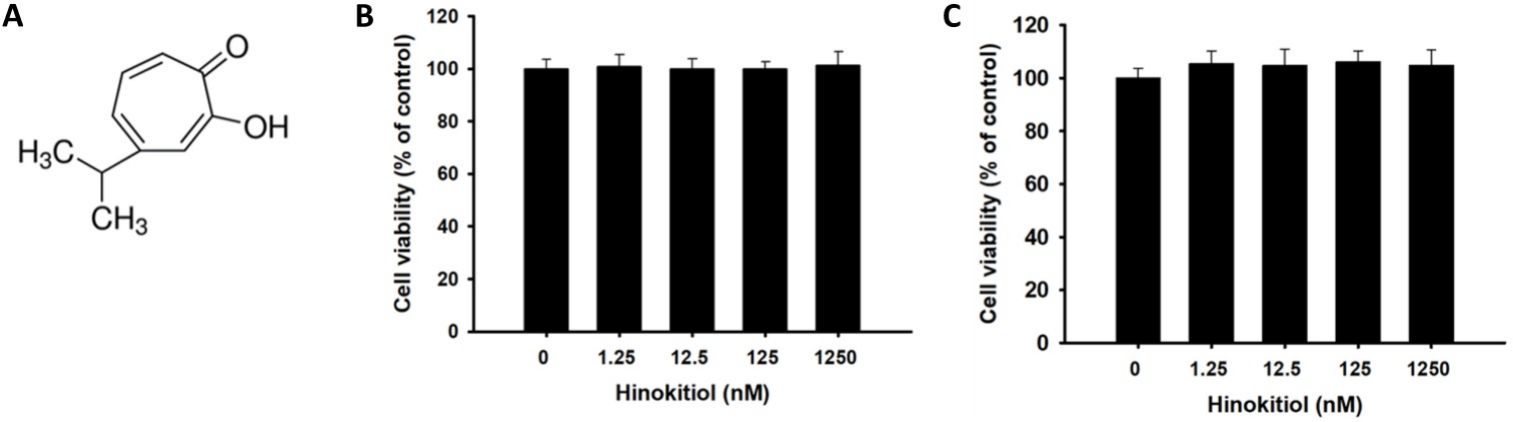
Effects of hinokitiol on tumor cell viability. (A) Chemical structure of hinokitiol. B16F10 (A) and CT26 (B) cells were treated with hinokitiol (0-1200 nM) for 16 h. The number of cell was measured by WST-8 assay.

The purpose of this study is to reveal the potential of hinokitiol as a chemosensitizing agent. Hinokitiol exerts a wide variety of biologic activities, including antimicrobial, antioxidant, and antimetastatic activities 10, 22. Hinokitiol, also known as β-Thujaplicin, is a natural tropolone derivative, an isopropyl side chain, and a seven-membered carbon ring (Fig. 1A). This study determined the optimal concentration for hinokitiol that can affect tumor cell biological activities without harming the cells because excessive concentration may lead to direct cell death, including those with normal function. As shown in Fig. 1, hinokitiol treatment at different concentrations (0-1250μM) did not significantly influence the tumor cell viability in this study.

### Intracellular accumulation of Rho-123 after hinokitiol treatment

**Figure 2:**
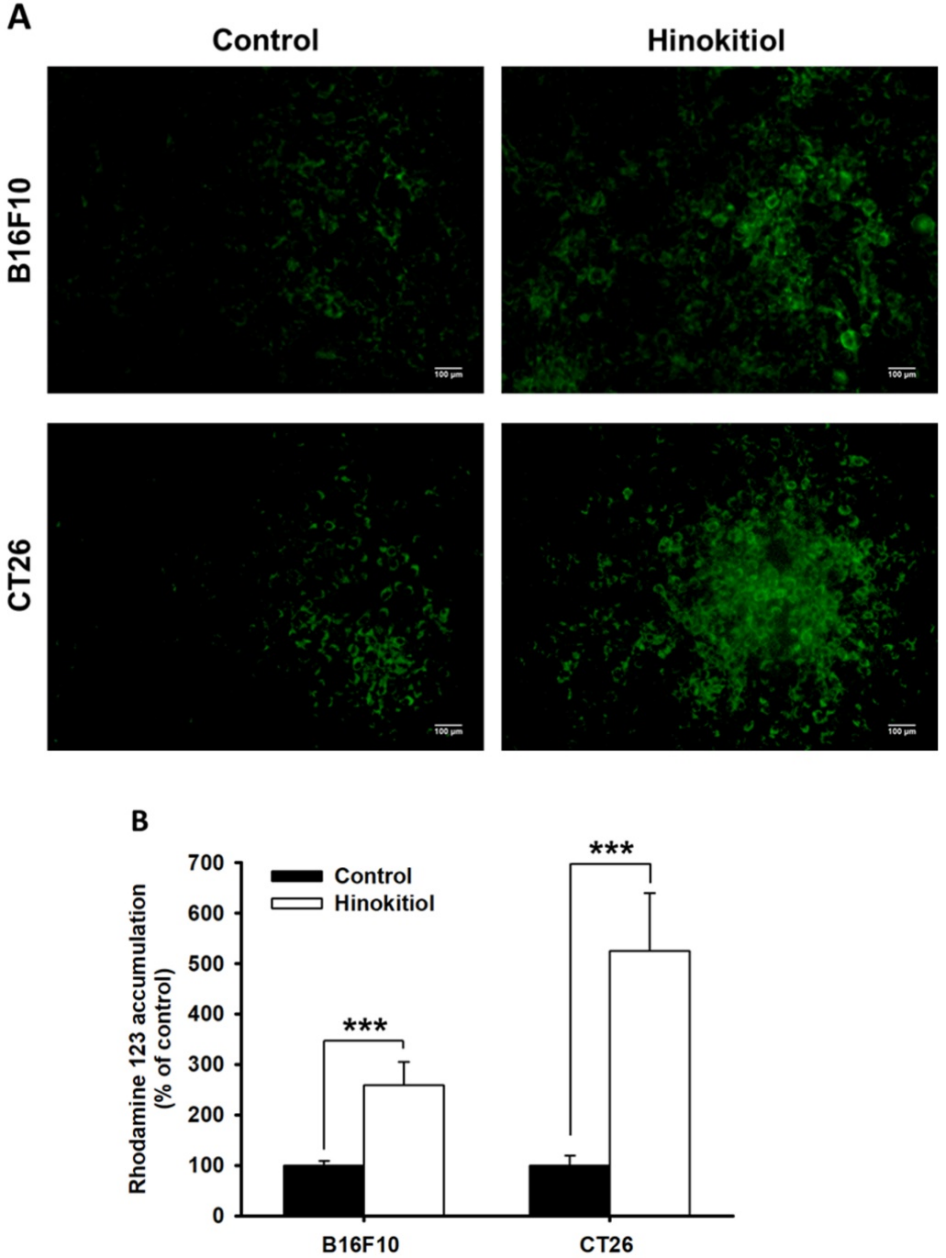
Intracellular accumulation of rhodamine 123 (Rho-123) in B16F10 and CT26 cells treated with hinokitiol. B16F10 and CT26 cells were treated with hinokitiol for 16 h, washed and incubated with culture medium for 2 h prior to addition of Rho-123. (A) The accumulation of Rho-123 in cells was analyzed using a fluorescence microscope. (B) The Rho-123 were determined from the fluorescence by using spectrofluorometer. *** *P <* 0.001.

The intracellular accumulation of fluorescent (Rho-123) showed the P-gp function in tumor cells 23. To analyze whether hinokitiol could influence P-gp transport activity after hinokitiol treatment in tumor cells, the intracellular accumulation of Rho-123 was measured 8. As shown in Fig. 2A, Rho-123 was detected at low levels at the nuclear periphery and in the cytoplasm of control cells, whereas it was detected exclusively in the cytoplasm of cells treated with hinokitiol. This phenomenon was observed in the two tumor cells tested (Fig. 2). By measuring the fluorescence values, the accumulation of Rho-123 was found to be significantly higher in the hinokitiol-treated cells than that in the control cells (Fig. 2B). These results indicate that hinokitiol may suppress P-gp transport or efflux activity leading to an accumulated Rho-123 in the cytoplasm of tumor cells.

### Hinokitiol reduced P-gp expression through P-AKT/ P-mTOR pathway

**Figure 3:**
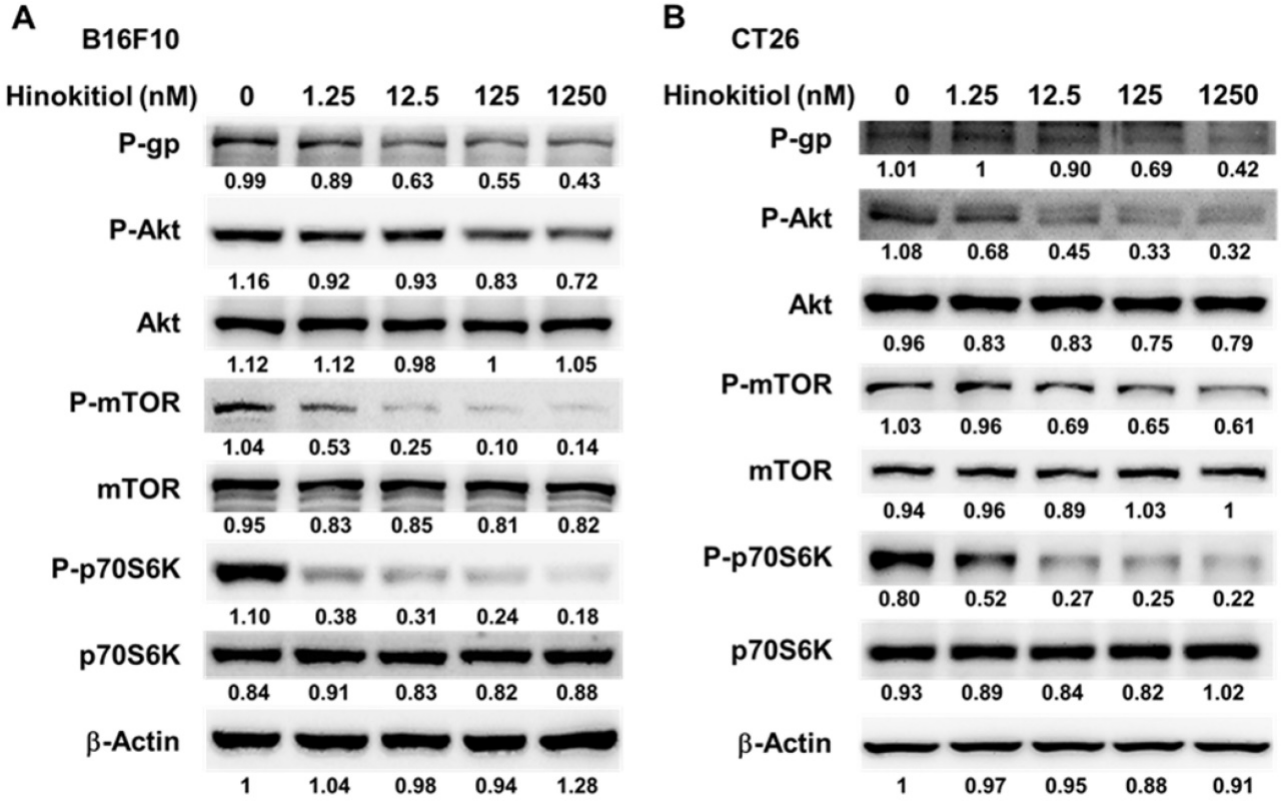
Hinokitiol regulated the expression of P-glycoprotein (P-gp), and AKT/mTOR signaling pathway. The expression of P-gp, AKT, mTOR, p70s6K was measured in B16F10 (A) and CT26 (B) cells by Western blotting analysis. The β-actin expression served as loading controls for and total protein. Each experiment was repeated three times with similar results.

**Figure 4:**
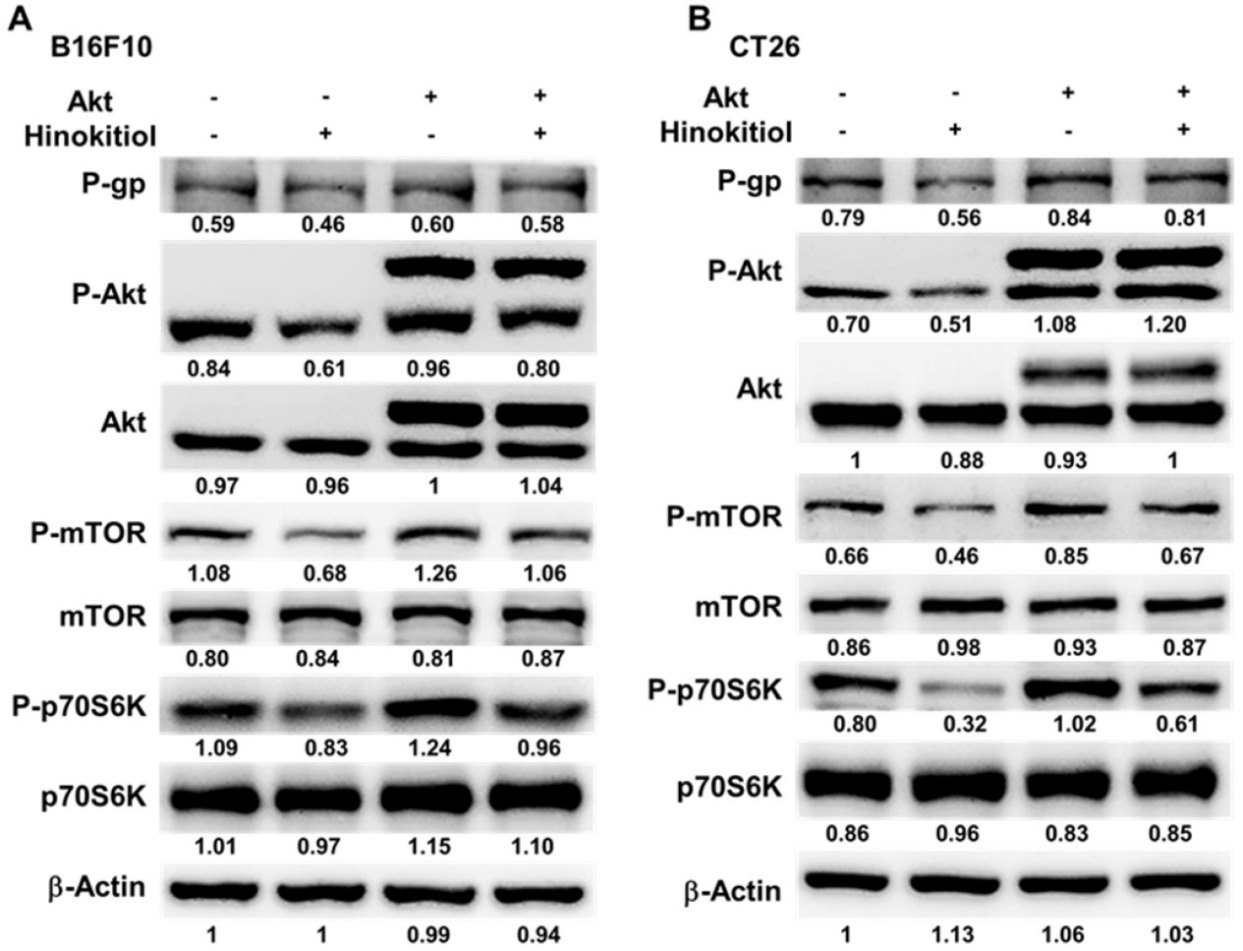
Constitutively active-AKT reduced the effects of hinokitiol on tumor cells. The B16F10 and CT26 cells were transfected with constitutively active AKT plasmid (3.5 μg) for 16 h prior to treatment with hinokitiol (1250 nM) or not for 16 h. The expression of P-glycoprotein (P-gp), AKT, mTOR, p70s6K protein in B16F10 (A) and CT26 cells (B) was determined. The β-actin expression served as loading controls for and total protein. Each experiment was repeated three times with similar results.

We used Western blotting analysis to study the correlation between P-gp and hinokitiol treatment. As shown in Fig. 3, the protein levels of P-gp were decreased after hinokitiol treatment. Furthermore, the phosphorylation of p70s6K induced by AKT/mTOR signaling pathway promotes P-gp protein expression 8. Hinokitiol significantly reduced the phosphorylation of AKT, mTOR, and p70s6K in B16F10 and CT26 cells in a dose dependent manner (Fig. 3A and B). These results demonstrated that inhibition of AKT/mTOR/p70s6K signaling pathway after hinokitiol treatment was associated with the reduction of P-gp expression. We further analyzed the effects of hinokitiol treatment on AKT/mTOR/p70s6K signaling pathway by transfection with constitutively active AKT plasmid. In AKT transfected cells, hinokitiol demonstrated a reduced suppression of mTOR/ p70s6K/P-gp expression (Fig. 4A and B). The results suggest that downregulation of AKT/mTOR signaling pathway is necessary for hinokitiol-mediated reduction of P-gp expression in tumor cells.

### Hinokitiol sensitizes tumor cells to 5-FU

**Figure 5:**
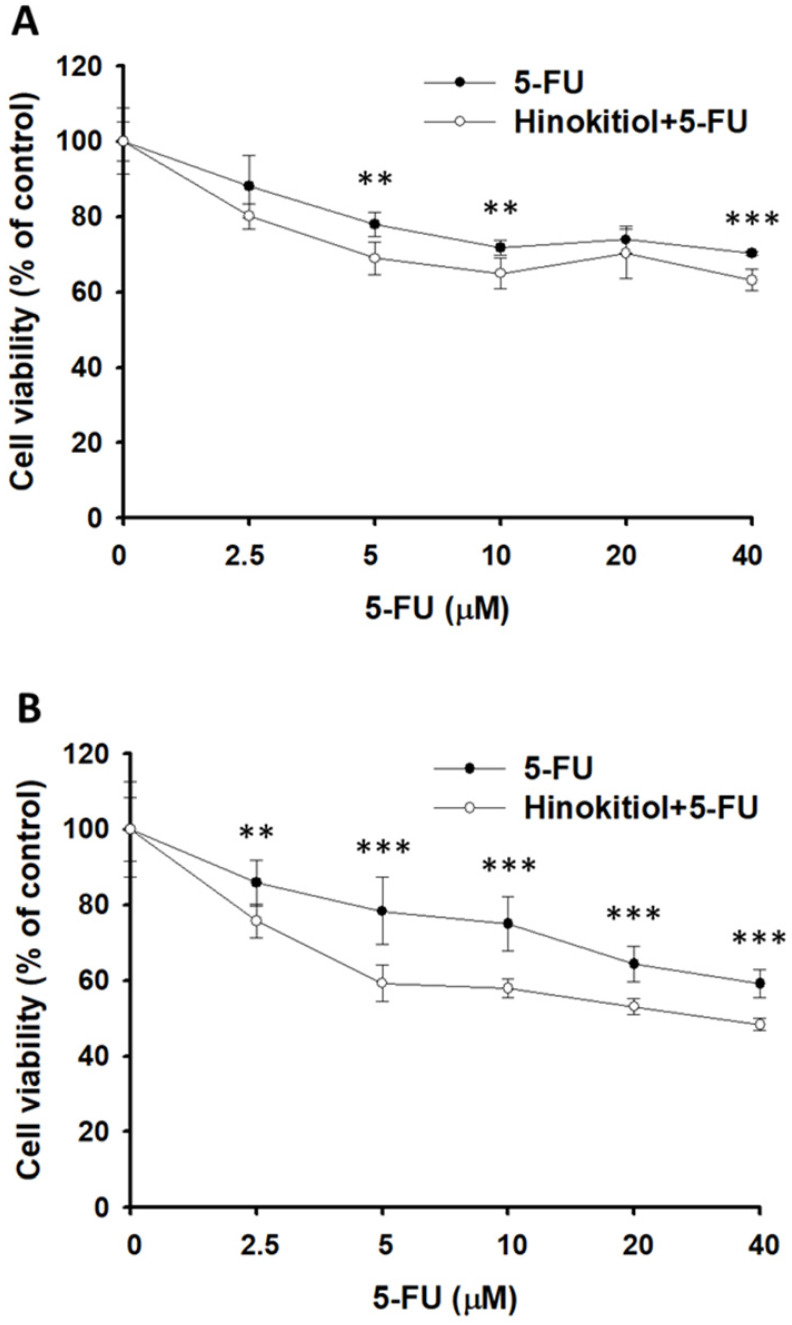
Hinokitiol-regulated P-glycoprotein (P-gp) expression in conjunction with 5-FU exerted cytotoxic effects on B16F10 and CT26 cells. Hinokitiol-treated (1250 nM), or control cells were exposed to 5-FU (0-40 µM) for 48 h followed by determination of their viability by the WST-8 assay in B16F10 (A) and CT26 (B) cells. Data are expressed as mean ± SD of hexaplicate determinations. ** *P <* 0.01; *** *P <* 0.001.

Furthermore, we examined the downregulation of P-gp expression after hinokitiol treatment whether it will enhance the sensitivity of tumor cells to 5-FU-induced cell death. Herein, the proliferation of tumor cell was measured after 5-FU, or hinokitiol combined with 5-FU treatment. The results show that the number of tumor cells were lowest when tumor cells were treated with hinokitiol + 5-FU (40 μM) compared with the control group (Fig. 5A and B). Treatment with 5-FU significantly enhanced cell death in hinokitiol-treated cells as compared to untreated cells. Collectively, these results indicate that hinokitiol may inhibit the proliferation of tumor cells through enhancing susceptibility of tumor cells to 5-FU.

### Hinokitiol in combination with 5-FU enhanced the antitumor activity *in vivo*

**Figure 6:**
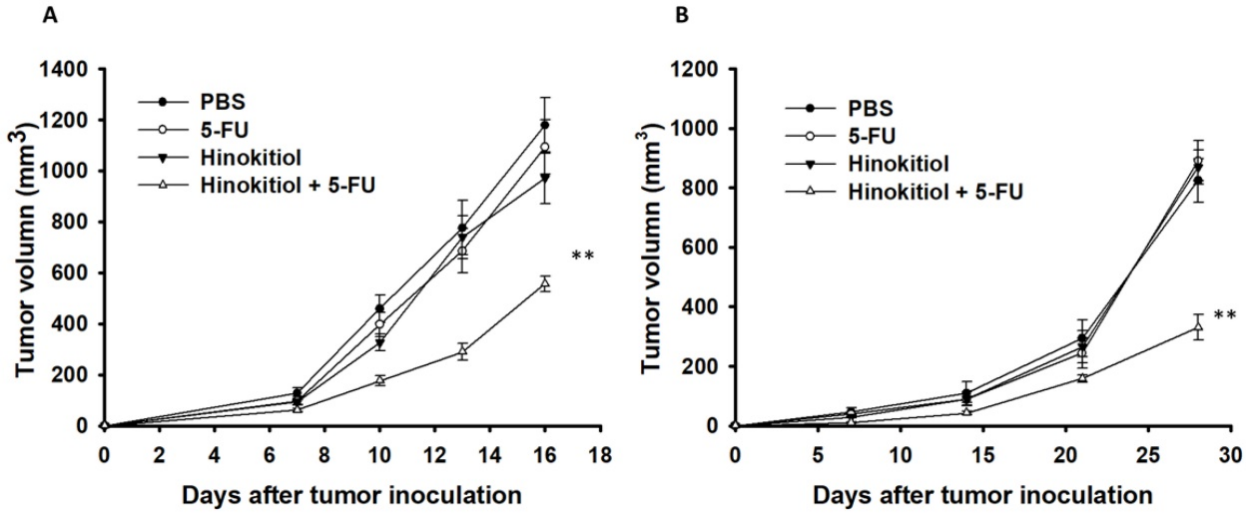
Additive antitumor effects of hinokitiol combined with 5-FU on subcutaneous B16F10 and CT26 tumors. Groups of 8 mice, that had been inoculated s.c. with B16F10 (A) and CT26 cells (B) at day 0, were treated intraperitoneal with hinokitiol (40 mg/kg) at day 7 followed by 5-FU (40 mg/kg) treatment at days 9, 11, and 13, or with either treatment alone. Vehicle control mice received PBS. Tumor volumes (mean ± SEM, n = 8) among different treatment groups were compared in mice bearing tumors. ** P < 0.01.

**Figure 7:**
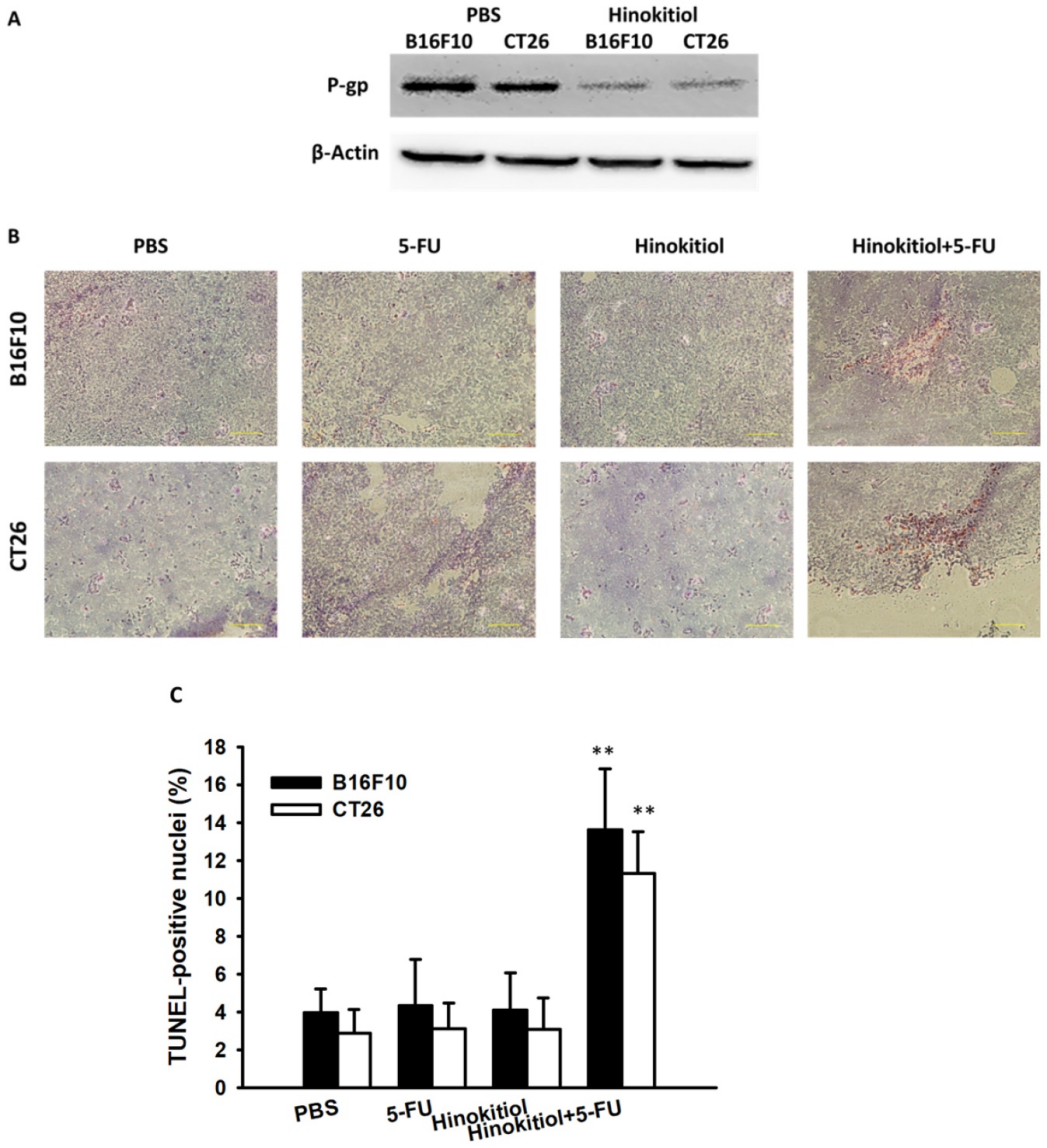
Increase in tumor cells undergoing apoptosis in tumor-bearing mice treated with hinokitiol in combination with 5-FU. Groups of 3 mice, that had been inoculated s.c. with B16F10 and CT26 cells (10^6^) at day 0, were treated intraperitoneally with hinokitiol (40mg kg) at day 7 followed by 5-FU (40 mg/kg) at days 9, 11, and 13, or with either treatment alone. Vehicle control mice received PBS. (A) Tumors were collected at day 15, and Western blotting assay was used to detect protein expression. (B) Tumors were excised at day 15, and TUNEL assay was used to detect apoptotic cells (× 200). (C) TUNEL-positive cells were counted from three fields of highest density of positive-stained cells in each section to determine the percentage of apoptotic cells (mean ± SEM, n =3). **, *P <* 0.01.

To confirm our *in vitro* findings, we performed *in vivo* experiments using tumor-bearing mice. Here, we evaluated the antitumor effects of hinokitiol in combination with 5-FU. Fig. 6 shows that hinokitiol and low-dose 5-FU groups did not cause substantial reduction of tumor growth in comparison with PBS-treated control mice and that the combination therapy, i.e., hinokitiol and 5-FU, significantly inhibited the tumor growth. The tumor volume of mice treated with the combo therapy was reduced by 52.79% in B16F10 model and 59.96% in CT26 model when compared with those in the PBS-treated group. The Western blotting assay provides the P-gp expression level in the tumor after hinokitiol treatment (Fig. 7A). Meanwhile, TUNEL assay was used for the histopathological examination of the tumor sections from tumor-bearing mice treated with PBS, hinokitiol or 5-FU alone, or in combination group. The TUNEL assay showed an increase in the number of cells undergoing apoptosis in the hinokitiol plus 5-FU-treated mice compared with the PBS-treated mice (Fig. 7B and C). Few signals were observed in the hinokitiol or low-dose 5-FU group. There was a 3.41~3.91-fold increase in the number of apoptotic cells induced by hinokitiol plus 5-FU compared with these in the control group (Fig. 7C). Taken together, hinokitiol combined with 5-FU demonstrated an additive antitumor effect.

## Discussion

The effects of the combinational treatment of hinokitiol and 5-FU *in vitro* and* in vivo* were determined using mouse melanoma and colon tumor models. The combination of hinokitiol and 5-FU inhibits tumor growth, suggesting that hinokitiol can increase the efficacy of 5-FU. However, the detailed mechanism of the combinational therapy remained uncertain. In this study, we used mouse tumor models to examine the potential mechanisms of hinokitiol-induced alteration of signaling and the consequences of the combination therapy specifically on AKT/mTOR signaling and the expression of multidrug resistance protein P-gp. Previously, we found that bacteria demonstrated the ability to decrease the expression of P-gp via posttranscriptional regulation 8. In the context of natural products, the downregulation of P-gp expression via inhibition of AKT/mTOR pathway using Ganoderma-derived fungal protein has also been demonstrated 24. In the present study, an increase in the amount of tumor cells undergoing apoptosis were observed in tumor sections from mice treated with hinokitiol + 5-FU. Our results have demonstrated that hinokitiol increases the antitumor effects of 5-FU via intracellular drug retention as a consequence of hinokitiol-mediated downregulation of P-gp expression.

A recent study demonstrated that hinokitiol treatment induces tumor cell death via sensitization of human lung cancers to TNF-related apoptosis-inducing ligand (TRAIL)-mediated apoptosis and suppression of X-linked inhibitor of apoptosis protein (XIAP) 15. Hinokitiol has also been shown to inhibit tumor cell proliferation, in part, by G1 cell cycle arrest characterized by downregulated pRb and Skp2 ubiquitin ligase, and an impaired Cdk2 kinase activity 25. Hinokitiol can directly induce tumor cell death through different pathways in other studies that were triggered after hinokitiol treatment. High intracellular cytotoxic drug concentration in tumor cells allows killing of tumor cells but a growing number of evidence show an increase in chemotherapy resistance on account of P-gp upregulation. For this reason, P-gp expression in tumor cells may influence the clinical response to chemotherapy 26. In our model, we showed that hinokitiol treatment at specified concentration suppresses expression of P-gp that corresponded to a substantial decrease of drug efflux activity. Our results show that hinokitiol-mediated regulation of P-gp in combination with 5-FU treatment may provide new insights for tumor therapy. 5-FU is one of the most commonly used drugs to treat cancers including breast, colon and some forms skin cancer 27. The major limiting factor in the use of 5-FU is the side effects in normal tissue including diarrhea, mouth ulcers, and hair thinning 28. The significant decrease in drug efflux activity following P-gp downregulation would allow the use of lower 5-FU concentration, thus decreasing the occurrence side effects.

In conclusion, the levels of phosphorylated AKT, mTOR, and p70s6K were significantly decreased in hinokitiol-treated cancer cells. This downmodulation of Akt signaling cascade after hinokitiol treatment resulted in the suppression of P-gp-mediated drug efflux activity that allowed the cytotoxic drug 5-FU to induce tumor cell death more efficiently. By taking advantage of the cytotoxicity effect of 5-FU and the pleiotropic drug effects of hinokitiol, i.e., P-gp downregulation, we suggest that hinokitiol can be utilized to reverse cancer drug resistance, particularly to 5-FU in the present model.
